# Second Trimester Fetal Cardiac Screening – Current Opinion

**DOI:** 10.1055/s-0043-1764492

**Published:** 2023-03-28

**Authors:** Nathalie Jeanne Bravo-Valenzuela, Alberto Borges Peixoto, Edward Araujo Júnior

**Affiliations:** 1 Department of Pediatrics, Pediatric Cardiology, Federal University of Rio de Janeiro, RJ, Rio de Janeiro, Brazil; 2 Gynecology and Obstetrics Service, Mário Palmério University Hospital, University of Uberaba, Uberaba, MG, Brazil; 3 Department of Obstetrics and Gynecology, Federal University of Triângulo Mineiro, Uberaba, MG, Brazil; 4 Department of Obstetrics, Paulista School of Medicine, Federal University of São Paulo, São Paulo, SP, Brazil; 5 Medical course, Municipal University of São Caetano do Sul, São Caetano, SP, Brazil

Congenital malformations are a major cause of miscarriage, perinatal mortality, prematurity, childhood death and disability worldwide. The well-being of each affected child with a birth defect depends primarily on which organ or body part is affected and how severely. The World Health Organization (WHO) estimates that approximately 295,000 newborns die each year as a result of congenital anomalies during the neonatal period. 1 The development of research in this area, the early diagnosis of birth defects, the promotion of prenatal screening programs, and the development of preventive public health strategies are important tools with great impact on the prognosis of these defects. The most common birth defects are cardiac anomalies, neural tube defects and trisomy 21. 1 2

The prevalence of congenital heart disease (CHD) has been increasing worldwide, especially in the last 15 years. 2 3 4 The literature shows that the incidence of CHD varies with the type of population studied, ranging from approximately 4 to 10-12 cases of CHD per 1000 live births. 4 5 6 7 8 In this scenario, for example, CHD may be twice as common in premature infants and up to five times more common in infants born to mothers with pregestational diabetes. 9 10 The Centers for Disease Control and Prevention (CDC) estimates that there are approximately 40 thousand new cases of CHD per year in the United States, and the WHO estimates that there are approximately 1 million cases of CHD per year. 1 11 In Brazil, according to the statistics of the Instituto Brasileiro de Geografia e Estatística (IBGE) in 2010, 10 the number of live births per year was approximately 3.2 million, and considering that 10 out of 1000 live births have CHD, we estimate that there are approximately 32 thousand new cases of CHD per year. 12 Cardiac anomalies are an important cause of perinatal death related to birth defects and are the leading cause of mortality in the population under 20 years of age due to non-communicable diseases. 13 These incidences highlight the importance of knowing the early diagnosis of congenital heart disease for its therapeutic management. It is also important to emphasize that the development of CHD is multifactorial, involving both genetic and environmental factors. 14 In this setting, prenatal sonographic cardiac screening, which involves obstetricians, radiologists, and fetal medicine subspecialists, is an important tool for suspecting cardiac defects in high-risk and especially low-risk pregnant women.

**Fig. 1.**
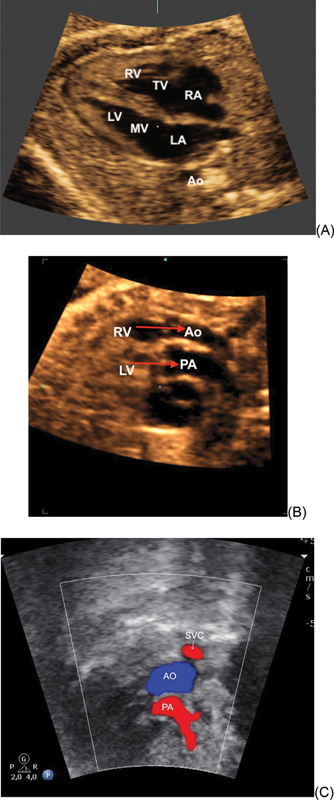
(A) Normal four-chamber view in a case of transposition of the great arteries. (B) Abnormal outflow tracts in the same fetus as in A. Note the parallel relationship of the great arteries and the ventriculoarterial discordance. (C) Abnormal three-vessel view and trachea in a case of tetralogy of Fallot with pulmonary atresia: note the small pulmonary artery (PA). The red color of the pulmonary artery and the blue color of the aorta show the retrograde flow of the PA due to the pulmonary atresia. LA: left atrium; LV: left ventricle; RA: right atrium; RV: right ventricle; MV: mitral valve; TV: tricuspid valve; Ao: aorta; PA: pulmonary artery; SVC: superior vena cava.

In light of the above, and considering that only 10% of fetuses with congenital heart disease present risk factors, ultrasound fetal cardiac screening programs have been designed to maximize the detection of cardiac malformations as an essential tool for the prenatal diagnosis of CHD, mainly in the evaluation of low-risk fetuses examined during routine ultrasound prenatal care. Furthermore, this approach allows the detection of fetuses with malformations and at risk for genetic syndromes, providing useful elements for parental counseling, prenatal obstetric management, multidisciplinary care, and also allows a more appropriate referral to perinatal cardiology (fetal echocardiography). In the late 1980s, the four-chamber (4C) view of the fetal heart became part of prenatal cardiac screening. The evaluation of the main anatomical features of the four heart chambers and the atrioventricular valves, as well as the assessment of the global size, axis and position of the heart, are the main elements of the 4C cardiac scan. However, only 30 to 48% of CHDs can be detected when prenatal screening is based on the visualization of the 4C view. 15 16 This is mainly due to the fact that major anomalies, such as conotruncal CHD, present normal imaging of the fetal heart in the 4C view ( Figure 1A ). Therefore, the diagnosis of such anomalies (transposition of the great vessels, tetralogy of Fallot, truncus arteriosus, and interrupted aortic arch) is missed when only the 4C view is used for cardiac ultrasound screening. In addition, this approach is inadequate for the detection of ventricular outflow tract obstruction and outlet septal defects ( Figures 1B e 1C ). 15 16 17 18

When outflow tract evaluation was added to the 4C view, the sensitivity of ultrasound screening for CHD increased from approximately 30-48% to 69-83%. 15 16 17 18 Subsequently, the upper mediastinal views [three-vessel (3V) and three-vessel with trachea (3VT) views] were added to the standard 4C and outflow views to further improve detection of CHD. 17 19 20 21 Thus, the diagnosis of vascular rings, aortic arch and ductal anomalies, and obstructive outflow tract lesions was optimized by the addition of the 3V and 3VT views ( Figure 1C ). Moreover, in this view, the measurement of the thymus-thoracic ratio is a viable and useful tool in fetuses with CHD associated with absent or hypoplastic thymus, as in CATCH-22q11deletions, 22 with a positive impact on reducing mortality associated with this malformation. Then, in 2013, the International Society of Ultrasound in Obstetrics and Gynecology (ISUOG) published a guideline describing that the views of the outflow tracts and the upper mediastinum should be added to the 4C view during the sonographic screening examination of the fetal heart. 17

In our opinion, the use of the ISUOG guidelines in the evaluation of the fetal heart during routine second trimester ultrasound is of paramount importance in the evaluation of the heart during routine second trimester fetal heart ultrasound in order to maximize the diagnosis of CHD. To achieve this goal, it is fundamental that the professional who performs the cardiac screening should be very well trained in the acquisition of fetal heart views (4C, outflow tracts, 3V and 3VT views) through practical teaching with hands-on approach in specialized centers. In addition, a real problem, even with well-trained examiners, is the lack of time due to the large number of patients, especially when the fetal position is unfavorable. In such cases, the acquisition of a cine loop of the fetal heart can be useful for more detailed off-line analysis. In fact, when the fetus is in a difficult position, an interesting suggestion is to ask the pregnant woman to go for a walk and return later or to schedule another day to complete the cardiac scan.

**Fig. 2.**
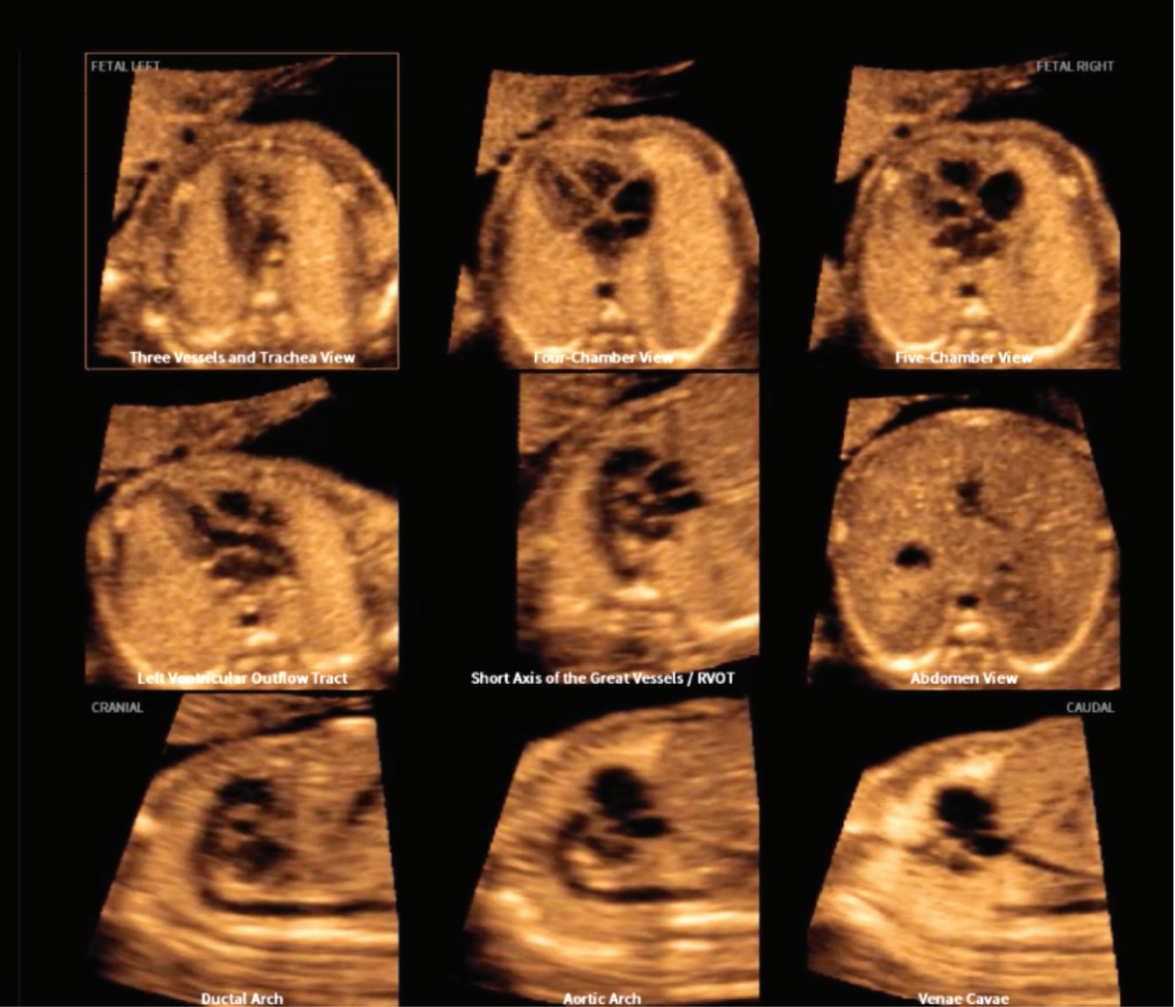
The Fetal Intelligent Navigation Echocardiography (FINE) method, known as "5D heart" or "5D", provides automatic reconstruction of 9 fetal heart views by acquiring one cycle of a sequence of ultrasound images of the four-chamber view.

In recent decades, advanced technologies such as three-dimensional (3D) and four-dimensional (4D) ultrasound with spatiotemporal image correlation (STIC) have been developed and made available, allowing more detailed sonographic images of the fetal heart when integrated with routine two-dimensional (2D) ultrasound imaging. 23 In addition, these images can be further analyzed (offline) and also sent online to an expert (perinatal cardiologist) to clarify questions. Currently, the automatic reconstruction of all fetal cardiac views recommended during the performance of a fetal echocardiogram can be automatically generated by the Fetal Intelligent Navigation Echocardiography (FINE) method, known as "5D heart". 22 23 This automatic reconstruction of 9 of the fetal cardiac views (3V, 3VT, four and five chambers, left and right ventricular outflow tracts, short axis of the great vessels, abdomen, aortic and ductal arches, and vena cava views) is generated from an acquisition of a sequence of ultrasound images of the 4C view ( Figure 2 ). More recently, ''artificial intelligence'' is a promising technological innovation that allows: 1- screening of cardiac anomalies by ultrasound by warning of possible fetal heart anomalies, allowing greater suspicion of the diagnosis of CHD by non-specialists and more accurate selection of cases to be referred for echocardiogram examination, 2- reduction of examination time by automatic reconstruction of cardiac views.

In conclusion, cardiac screening is crucial to increase the detection rate of CHD, especially in the low-risk population where fetal echocardiography is not formally indicated. In this context, the adequate training of the professional performing the fetal cardiac screening should focus on practical teaching of the acquisition of the outflow tract, 3V and 3VT in addition to the 4C view. More successful fetal cardiac ultrasound screening has a positive impact on the management of CHD and its associated mortality.
